# Endovascular embolization by a magnetic microfiberbot

**DOI:** 10.1093/nsr/nwae117

**Published:** 2024-04-04

**Authors:** Shuning Zhang, Wenlong Yang, Junbo Ge

**Affiliations:** Department of Cardiology, Zhongshan Hospital, Fudan University, Shanghai Institute of Cardiovascular Diseases, China; Key Laboratory of Viral Heart Diseases, National Health Commission, China; Key Laboratory of Viral Heart Diseases, Chinese Academy of Medical Sciences, China; National Clinical Research Center for Interventional Medicine, China; Institutes of Biomedical Sciences, Fudan University, China; Department of Cardiology, Zhongshan Hospital, Fudan University, Shanghai Institute of Cardiovascular Diseases, China; Key Laboratory of Viral Heart Diseases, National Health Commission, China; Key Laboratory of Viral Heart Diseases, Chinese Academy of Medical Sciences, China; National Clinical Research Center for Interventional Medicine, China; Institutes of Biomedical Sciences, Fudan University, China; Department of Cardiology, Zhongshan Hospital, Fudan University, Shanghai Institute of Cardiovascular Diseases, China; Key Laboratory of Viral Heart Diseases, National Health Commission, China; Key Laboratory of Viral Heart Diseases, Chinese Academy of Medical Sciences, China; National Clinical Research Center for Interventional Medicine, China; Institutes of Biomedical Sciences, Fudan University, China

The evolving landscape of embolization therapy for treating cerebral aneurysms and brain tumors represents a pivotal stride towards enhancing the efficacy and safety of minimally invasive treatments [1]. Conventional endovascular embolization methods [2], characterized by the manual maneuvering of catheters through the vascular system, present notable limitations in steerability and precision, compounded by the potential health risks associated with prolonged exposure to radiation for surgeons.

**Figure 1.**
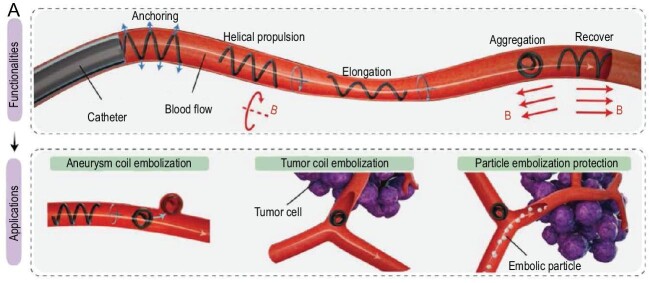
Magnetic soft microfiberbots for robotic embolization.

In a significant publication in *Science Robotics*, a collaborative team led by Professors Jianfeng Zang, Guangming Tao and Guang-Zhong Yang has pioneered a breakthrough in robotic embolization [3]. Their work introduces magnetic soft microfiber robots (Fig. 1), promising to revolutionize this field by directly tackling existing challenges with their innovative design. Microfiberbots, with their helical geometry and customizable diameter, introduce an innovative approach to navigating the tortuous and confined pathways of the vascular system. Their design allows for compatibility with existing catheterization techniques, thereby enhancing clinical applicability. The application of remote magnetic fields to steer these microfiberbots within blood vessels signifies a monumental leap in achieving targeted delivery of embolic agents with unprecedented precision and control. This remotely controllable operation not only augments the steerability and maneuverability of embolization procedures but also minimizes the radiation exposure to surgeons, fostering a safer procedural environment.

Conventional fiber fabrication methodologies, such as electrospinning,

wet-spinning and melt-spinning, commonly involve single fluidic flow of precursor materials before the final formation. The multimaterial fiber thermal drawing methodology [4,5], adopted to fabricate the magnetic microfibers, enables the continuous co-flow of the inner magnetic composite core and outer sacrificial thermoplastic polymer cladding. The outer polymer layer permits not only the consistent magnetic fiber fabrication but also the ultrathin features via the effective suppression of fluidic instability [6]. Moreover, such multimaterial fibers can convey diverse operational functionalities such as laser transmission [7], electrical ablation and microfluidic delivery [8,9]. Further incorporation of machine intelligence into the system permits fiberbots that can navigate, steer, perceive, learn and solve during interventions [10].

The significance of this work lies not only in its immediate application to the field of embolization but also in its potential to redefine the parameters of what is achievable in minimally invasive surgical treatments. The enhanced steerability, reliable maneuverability and on-demand shape-morphing capabilities of the

microfiberbots facilitate precise occlusion of blood flow to targeted lesions, offering a promising adjunct or alternative to traditional catheter-based embolization techniques. The demonstrated *in vitro* and *in vivo* efficacy of these microfiberbots in embolizing aneurysms and tumors underlines the practical viability and potential transformative impact of this technology on patient outcomes and surgical safety.

However, the journey from bench to bedside is fraught with challenges that need to be meticulously addressed to realize the full potential of this technology. The considerations for clinical translation highlighted in the article, including the customization of the microfiberbot's diameter for varying aneurysm sizes, the biocompatibility of materials, and the development of advanced positioning and tracking systems, underscore the multifaceted nature of bringing such innovations to clinical practice. Moreover, the exploration of strategies to expedite thrombus formation, such as the coating of microfiberbots with thrombin or iron oxide nanoparticles for radio frequency (RF)-induced hyperthermia, illustrates the ongoing efforts to enhance the efficacy and efficiency of embolization procedures.

In conclusion, while the development of magnetic soft microfiber robots represents a groundbreaking advancement in the field of robotic embolization, the realization of their full clinical potential necessitates further research and innovation. The exploration of biocompatible materials, precise control mechanisms and efficient thrombus formation techniques will be critical in overcoming the existing barriers to clinical adoption. As this technology matures, it holds the promise of revolutionizing the treatment of cerebral aneurysms and brain tumors, ushering in a new era of safer, more precise and minimally invasive therapeutic interventions.
